# Bicyclo­[2.2.1]hept-2-en-7-yl 4-bromo­benzoate

**DOI:** 10.1107/S1600536812027882

**Published:** 2012-06-27

**Authors:** Barry A. Lloyd, Atta M. Arif

**Affiliations:** a Department of Chemistry, Weber State University, Ogden, Utah 84403, USA; b Department of Chemistry, University of Utah, Salt Lake City, Utah 84112, USA

## Abstract

The structure of the title compound, C_14_H_13_BrO_2_, which contains a norbornenyl group and a 4-bromo­benzoate ester at the single C-atom bridge, has been redetermined [see McDonald & Trotter (1965 ▶). *Acta Cryst.* **19**, 456–463] to modern standards to establish high-precision geometrical data to compare with norbornyl and other tetra­cyclic 4-bromo­benzoates. Possible structural evidence is sought to help explain solvolytic reactivities.

## Related literature

For the previous structure determination of the title compound, see: McDonald & Trotter (1965 ▶). For a discussion, see: Coots (1983 ▶); Lloyd *et al.* (1995 ▶). For an analogous *p*-nitro­benzoate structure, see: Jones *et al.* (1992 ▶). For related tetra­cyclic 4-bromo­benzoate structures, see: Lloyd *et al.* (2000 ▶) and references therein. For a theoretical discussion, solvolysis rates and mol­ecular orbital calculations, see: Chow (1998 ▶). For further synthetic details, see: Coots (1983 ▶); Lloyd *et al.* (1993 ▶).
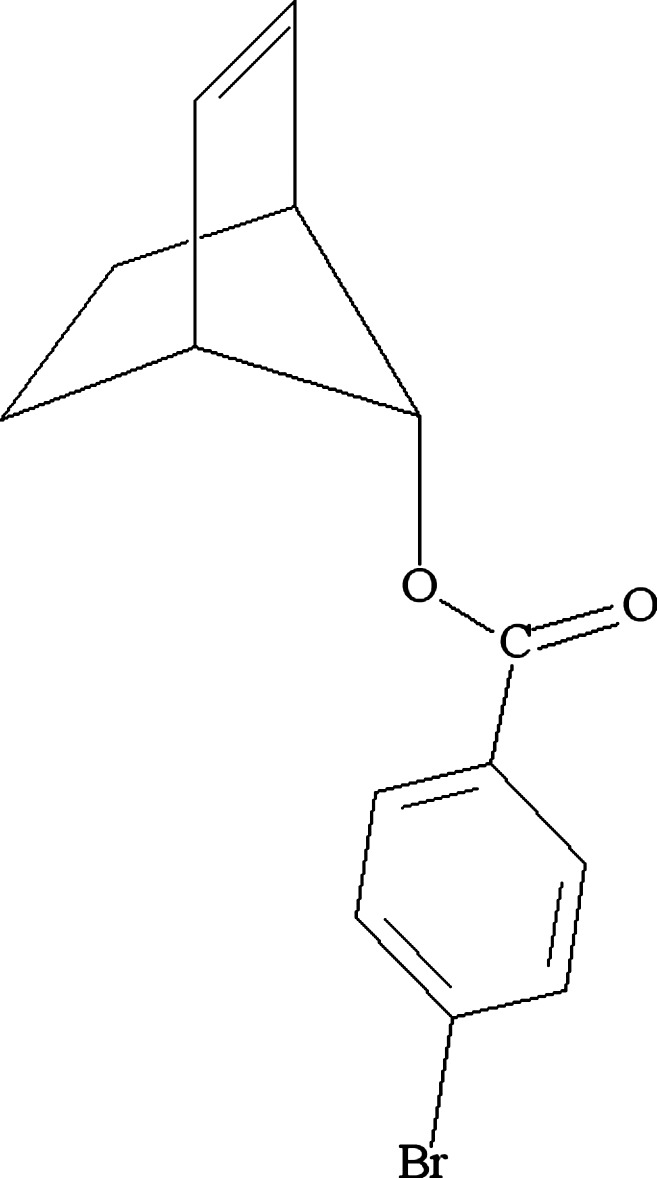


## Experimental

### 

#### Crystal data

C_14_H_13_BrO_2_
*M* *_r_* = 293.15
Monoclinic, 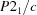

*a* = 14.0633 (2) Å
*b* = 10.0488 (1) Å
*c* = 8.6668 (1) Å
β = 100.0718 (7)°
*V* = 1205.91 (3) Å^3^
*Z* = 4
Mo *K*α radiation
μ = 3.40 mm^−1^
*T* = 150 K
0.35 × 0.33 × 0.30 mm

#### Data collection

Nonius KappaCCD diffractometer
Absorption correction: multi-scan (*DENZO-SMN*; Otwinowski & Minor, 1997 ▶) *T*_min_ = 0.383, *T*_max_ = 0.429
5345 measured reflections
2754 independent reflections
2509 reflections with *I* > 2σ(*I*)
*R*_int_ = 0.011

#### Refinement

*R*[*F* ^2^ > 2σ(*F* ^2^)] = 0.021
*wR*(*F* ^2^) = 0.051
*S* = 1.05
2754 reflections
207 parameters
All H-atom parameters refined
Δρ_max_ = 0.37 e Å^−3^
Δρ_min_ = −0.31 e Å^−3^

### 

Data collection: *COLLECT* (Nonius, 1998 ▶); cell refinement: *DENZO-SMN* (Otwinowski & Minor, 1997 ▶); data reduction: *DENZO-SMN*; program(s) used to solve structure: *SIR97* (Altomare *et al.*, 1999 ▶); program(s) used to refine structure: *SHELXL97* (Sheldrick, 2008 ▶); molecular graphics: *WinGX* (Farrugia, 1999 ▶), *ORTEP-3* (Farrugia, 1997 ▶) and *PLATON* (Spek, 2009 ▶); software used to prepare material for publication: *publCIF* (Westrip, 2010 ▶).

## Supplementary Material

Crystal structure: contains datablock(s) I, global. DOI: 10.1107/S1600536812027882/hb6776sup1.cif

Supplementary material file. DOI: 10.1107/S1600536812027882/hb6776Isup2.mol

Structure factors: contains datablock(s) I. DOI: 10.1107/S1600536812027882/hb6776Isup3.hkl

Supplementary material file. DOI: 10.1107/S1600536812027882/hb6776Isup4.cml

Additional supplementary materials:  crystallographic information; 3D view; checkCIF report

## Figures and Tables

**Fig. 1.**
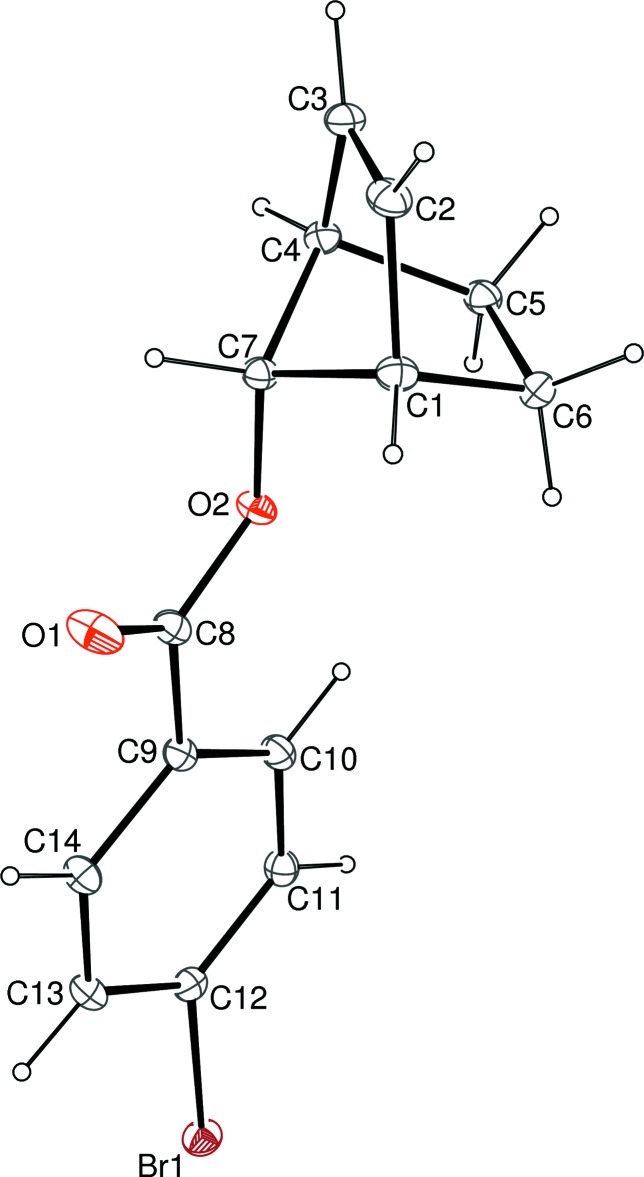
The molecular structure of the title compound, showing 50% displacement ellipsoids.

**Fig. 2.**
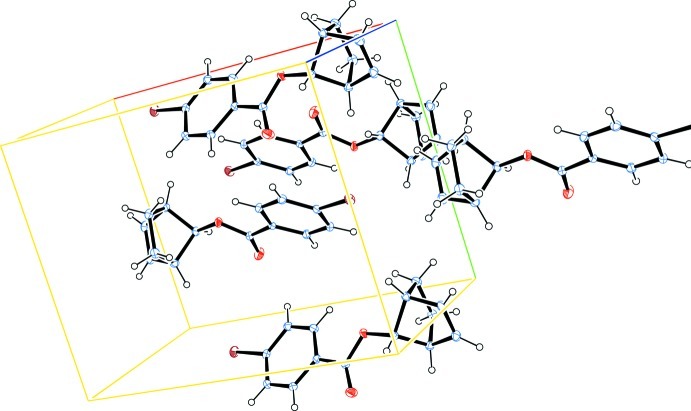
Packing diagram for the title compound.

**Fig. 3.**
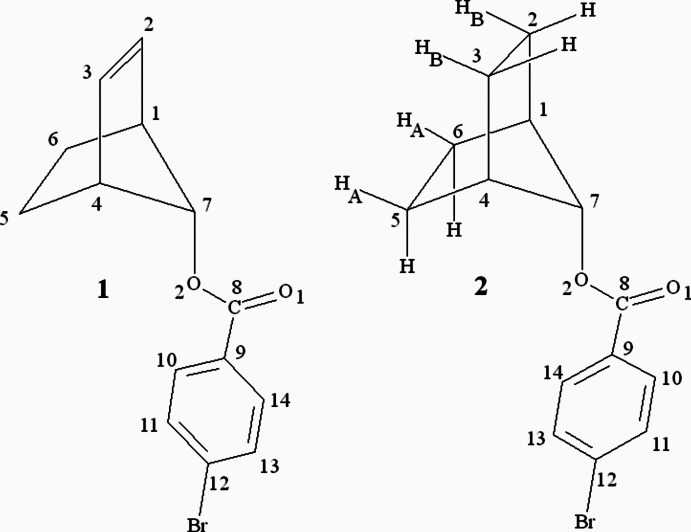
Compounds 1 and 2.

## supplementary crystallographic information

### Comment

Considerably improved precision is obtained for the present, low temperature structure of the title compound 1 over earlier structures. An *ORTEP-*3 drawing of 1 is shown in Fig. 1, and a cell packing diagram is shown in Fig. 2.

No nonhydrogen atom intermolecular contacts exist shorter than van der Waals radii sums. The closest contacts are shown in Table 1. Structure 1 least squares planes are defined as C1—C7—C4 (plane 1), C2—C3—C4—C5 (plane 2), C1—C6—C5—C4 (plane 3) and H2—C2—C3—H3 (plane 4). Structure 1 interplanar angles are compared to those of norbornyl structure 2 in Table 2.

The 2:4 angle shows that C2 and C3 are pyramidalized similarly as in other norbornenyl containing 4-bromobenzoate structures. The larger 1:2 and smaller 1:3 angle in 1*versus*2 may be a consequence of substituting an etheno bridge for an ethano bridge. The C1—C2, C2=C3, and C3—C4 bonds are shorter in 1*versus*2 as expected, but C1—C7 and C4—C7 are longer in 1 than in 2. These longer bonds possibly compensate for what might otherwise be even closer C2···C7 and C3···C7 intramolecular contacts in 1 (Table 3). A wider 1:2 angle in 1*versus*2 should also help relieve these contacts. Norbornenyl group bond lengths are all longer by 0.010 to 0.032 Å for structure 1*versus* the 293 K *p*-nitrobenzoate ester (Jones *et al.*,1992). The C7—O2 bond length, 1.445 (2) Å, is shorter than in the *p*-nitrobenzoate, 1.458 (3) Å, but is virtually the same as that of 2, 1.447 (2) Å, and gives no indication of the huge solvolytic reactivity difference between 1 and 2 derivatives.

### Experimental

*Anti*-7-norbornenyl 4-bromobenzoate (title compound 1) was prepared (Coots, 1983) from *anti*-7-norbornenol, which was made from 7-norbornenone reduction (Lloyd *et al.*, 1993, and references therein). In 25 ml of freshly distilled (from KOH under N_2_) dry pyridine was dissolved 0.700 g of sublimed (373 K, 1600 Pa) *anti*-7-norbornenol and 1.80 g of sublimed (373 K, 7 Pa) 4-bromobenzoyl chloride was added with stirring. The mixture was heated to 373 K for 5 min and set in a refrigerator overnight. The mixture was poured into 100 ml of cold water, and extracted three times with 100 ml of ether. Combined ether extracts were washed with cold 10% H_2_SO_4_ solution, saturated NaHCO_3_ solution, water, and finally saturated brine solution. The ether mixture was filtered, and ether was removed on a rotary evaporator (333 K maximum, 1600 Pa). Crude 1 residue was recrystallized from hexane yielding 1.21 g (65.0% yield) of 1. Sublimation (343 K, 1 Pa) further purified 1: mp 345–346 K. ^1^H NMR (CDCl_3_, 90 MHz): δ 1.10–1.14 (2 H, AB q, *J*= 4 Hz), 1.85 (2 H, m), 2.89 (2 H, m), 4.58 (1 H, bs), 6.08 (2 H, m), 7.56 (2 H, d, *J* = 9 Hz), 7.87 (2 H, d, *J* = 9 Hz), infrared (CCl_4_): 3066, 2980, 2878, 1723, 1597, 1485, 1396, 1308, 1275, 1118, 1088, 1011, 854, 760 cm^-1^. Anal. Calcd for C_14_H_13_BrO_2_: C 57.36, H 4.47. Found: C 57.11, H 4.49.

A 63.5 mg sample of 1 was dissolved by warming in 1.2 ml of freshly distilled absolute ethanol in a 10 ml beaker. The beaker was covered with aluminium foil, secured by a rubber band, and a pinhole was made in the foil for slow evaporation. About half of the ethanol had evaporated after 40 h at 296 K. A few small seed crystals were then added, which did not visibly dissolve. Crystal clumps began to grow. After 5 d total, about 0.2 ml of ethanol remained, and crystals were filtered out. One of these crystals was selected for X-ray analysis.

### Refinement

A colorless prism shaped crystal 0.35 × 0.33 × 0.30 mm in size was mounted on a glass fiber with traces of viscous oil and then transferred to a Nonius KappaCCD diffractometer equipped with Mo Kα radiation (λ = 0.71073 Å). Ten frames of data were collected at 150 (1) K with an oscillation range of 1 °/frame and an exposure time of 20 sec/frame (Nonius, 1998). Indexing and unit cell refinement based on all observed reflections from those ten frames, indicated a monoclinic *P* lattice. A total of 5345 reflections (Θ_max_ = 27.46°) were indexed, integrated and corrected for Lorentz, polarization and absorption effects using *DENZO– SMN* and *SCALEPAC* (Otwinowski & Minor, 1997). Post refinement of the unit cell gave a = 14.0633 (2) Å, b = 10.04880 (10) Å, c = 8.66680 (10) Å, β =100.0718 (7)°, and V = 1205.91 (3) Å^3^. Axial photographs and systematic absences were consistent with the compound having crystallized in the monoclinic space group *P*2_1_/*c*.

The structure was solved by a combination of direct and heavy atom methods using *SIR97* (Altomare *et al.*, 1999). All of the non-hydrogen atoms were refined with anisotropic displacement coefficients. Hydrogen atoms were located and refined isotropically using *SHELXL97* (Sheldrick, 2008). The weighting scheme employed was w = 1/[σ^2^(F_o_^2^) + (0.0214P)^2^ + 0.7334P] where P = (F_o_^2^ + 2F_c_^2^) /3. The refinement converged to R1 = 0.0211, wR2 = 0.0494, and S = 1.052 for 2509 reflections with I > 2σ(I), and R1 = 0.0245, wR2 = 0.0508, and S = 1.052 for 2754 unique reflections and 207 parameters, where R1 = Σ (|| F_o_ | – |F_c_ ||) / Σ |F_o_|, wR2 = [Σ(*w*(F_o_^2^ – F_c_^2^)2) / Σ(F_o_^2^)^2^]^1/2^, and S = Goodness-of-fit on F^2^ = [Σ (w(F_o_^2^ – F_c_^2^)^2^ / (n-p)] ^1/2^, n is the number of reflections and p is the number of parameters refined. The maximum Δ/σ in the final cycle of the least-squares was 0.002, and the residual peaks on the final difference-Fourier map ranged from -0.311 to 0.365 e/Å^3^. The structure was analyzed with *WinGX* (Farrugia, 1999) and *PLATON* (Spek, 2009).

### Crystal data

**Table:**

C_14_H_13_BrO_2_	*F*(000) = 592
*M**_r_* = 293.15	*D*_x_ = 1.615 Mg m^−^^3^
Monoclinic, *P*2_1_/*c*	Mo *K*α radiation, λ = 0.71073 Å
Hall symbol: -P 2ybc	Cell parameters from 2911 reflections
*a* = 14.0633 (2) Å	θ = 1.0–27.5°
*b* = 10.0488 (1) Å	µ = 3.40 mm^−^^1^
*c* = 8.6668 (1) Å	*T* = 150 K
β = 100.0718 (7)°	Prism, colourless
*V* = 1205.91 (3) Å^3^	0.35 × 0.33 × 0.30 mm
*Z* = 4	

### Data collection

**Table:**

Nonius KappaCCD diffractometer	2754 independent reflections
Radiation source: fine-focus sealed tube	2509 reflections with *I* > 2σ(*I*)
Graphite monochromator	*R*_int_ = 0.011
Phi and ω scan	θ_max_ = 27.5°, θ_min_ = 2.9°
Absorption correction: multi-scan (*DENZO-SMN*; Otwinowski & Minor, 1997)	*h* = −18→18
*T*_min_ = 0.383, *T*_max_ = 0.429	*k* = −13→12
5345 measured reflections	*l* = −11→11

### Refinement

**Table:**

Refinement on *F*^2^	Secondary atom site location: difference Fourier map
Least-squares matrix: full	Hydrogen site location: inferred from neighbouring sites
*R*[*F*^2^ > 2σ(*F*^2^)] = 0.021	All H-atom parameters refined
*wR*(*F*^2^) = 0.051	*w* = 1/[σ^2^(*F*_o_^2^) + (0.0214*P*)^2^ + 0.7334*P*] where *P* = (*F*_o_^2^ + 2*F*_c_^2^)/3
*S* = 1.05	(Δ/σ)_max_ = 0.002
2754 reflections	Δρ_max_ = 0.37 e Å^−^^3^
207 parameters	Δρ_min_ = −0.31 e Å^−^^3^
0 restraints	Extinction correction: *SHELXL97* (Sheldrick, 2008), Fc^*^=kFc[1+0.001xFc^2^λ^3^/sin(2θ)]^-1/4^
Primary atom site location: structure-invariant direct methods	Extinction coefficient: 0.0136 (7)

### Special details

**Table:**

Experimental. The program *DENZO-SMN* (Otwinowski & Minor, 1997) uses a scaling algorithm which effectively corrects for absorption effects. High redundancy data were used in the scaling program hence the 'multi-scan' code word was used. No transmission coefficients are available from the program (only scale factors for each frame). The scale factors in the experimental table are calculated from the 'size' command in the *SHELXL-97* input file.
Geometry. All e.s.d.'s (except the e.s.d. in the dihedral angle between two l.s. planes) are estimated using the full covariance matrix. The cell e.s.d.'s are taken into account individually in the estimation of e.s.d.'s in distances, angles and torsion angles; correlations between e.s.d.'s in cell parameters are only used when they are defined by crystal symmetry. An approximate (isotropic) treatment of cell e.s.d.'s is used for estimating e.s.d.'s involving l.s. planes.
Refinement. Refinement of *F*^2^ against ALL reflections. The weighted *R*-factor *wR* and goodness of fit *S* are based on *F*^2^, conventional *R*-factors *R* are based on *F*, with *F* set to zero for negative *F*^2^. The threshold expression of *F*^2^ > 2σ(*F*^2^) is used only for calculating *R*-factors(gt) *etc*. and is not relevant to the choice of reflections for refinement. *R*-factors based on *F*^2^ are statistically about twice as large as those based on *F*, and *R*- factors based on ALL data will be even larger.

### Fractional atomic coordinates and isotropic or equivalent isotropic displacement parameters (Å^2^)

**Table:**

	*x*	*y*	*z*	*U*_iso_*/*U*_eq_	
Br1	0.231948 (11)	0.573233 (17)	0.252195 (18)	0.02573 (7)	
O1	0.65735 (9)	0.74458 (13)	0.02284 (16)	0.0335 (3)	
O2	0.70549 (8)	0.56513 (11)	0.17202 (13)	0.0208 (2)	
C1	0.86438 (11)	0.67318 (16)	0.27173 (19)	0.0211 (3)	
C2	0.96258 (12)	0.64248 (18)	0.2314 (2)	0.0251 (3)	
C3	0.96191 (11)	0.51593 (18)	0.18542 (18)	0.0240 (3)	
C4	0.86326 (11)	0.45889 (16)	0.19263 (18)	0.0201 (3)	
C5	0.85573 (12)	0.44703 (16)	0.36921 (18)	0.0221 (3)	
C6	0.85694 (12)	0.59406 (17)	0.42354 (19)	0.0232 (3)	
C7	0.80342 (11)	0.58488 (15)	0.14674 (18)	0.0189 (3)	
C8	0.63967 (11)	0.65365 (15)	0.10386 (18)	0.0203 (3)	
C9	0.54164 (11)	0.62660 (15)	0.13984 (17)	0.0180 (3)	
C10	0.52464 (12)	0.53263 (16)	0.25011 (18)	0.0214 (3)	
C11	0.43223 (12)	0.51475 (16)	0.28209 (18)	0.0218 (3)	
C12	0.35770 (11)	0.59175 (15)	0.20240 (17)	0.0193 (3)	
C13	0.37227 (11)	0.68339 (16)	0.08950 (19)	0.0219 (3)	
C14	0.46492 (11)	0.69994 (16)	0.05865 (18)	0.0213 (3)	
H1	0.8462 (13)	0.7672 (19)	0.273 (2)	0.024 (5)*	
H2	1.0163 (16)	0.701 (2)	0.248 (2)	0.036 (5)*	
H3	1.0125 (16)	0.463 (2)	0.159 (2)	0.034 (5)*	
H4	0.8436 (14)	0.380 (2)	0.131 (2)	0.028 (5)*	
H5A	0.9080 (15)	0.3962 (19)	0.426 (2)	0.026 (5)*	
H5B	0.7950 (15)	0.4041 (19)	0.381 (2)	0.027 (5)*	
H6A	0.9089 (14)	0.615 (2)	0.506 (2)	0.026 (5)*	
H6B	0.7984 (15)	0.617 (2)	0.459 (2)	0.027 (5)*	
H7	0.8006 (13)	0.6132 (18)	0.037 (2)	0.017 (4)*	
H14	0.4765 (15)	0.761 (2)	−0.018 (2)	0.033 (5)*	
H13	0.3204 (15)	0.731 (2)	0.033 (2)	0.029 (5)*	
H11	0.4224 (15)	0.452 (2)	0.359 (2)	0.030 (5)*	
H10	0.5770 (16)	0.482 (2)	0.304 (2)	0.036 (5)*	

### Atomic displacement parameters (Å^2^)

**Table:**

	*U*^11^	*U*^22^	*U*^33^	*U*^12^	*U*^13^	*U*^23^
Br1	0.01967 (10)	0.03112 (11)	0.02766 (10)	−0.00351 (6)	0.00762 (6)	−0.00290 (6)
O1	0.0210 (6)	0.0299 (7)	0.0502 (8)	0.0011 (5)	0.0083 (5)	0.0176 (6)
O2	0.0139 (5)	0.0219 (6)	0.0261 (5)	0.0013 (4)	0.0024 (4)	0.0042 (4)
C1	0.0182 (7)	0.0185 (7)	0.0269 (8)	−0.0005 (6)	0.0048 (6)	−0.0018 (6)
C2	0.0170 (7)	0.0291 (9)	0.0296 (8)	−0.0016 (7)	0.0048 (6)	0.0040 (7)
C3	0.0184 (8)	0.0313 (9)	0.0236 (8)	0.0053 (7)	0.0070 (6)	0.0031 (7)
C4	0.0200 (7)	0.0193 (8)	0.0212 (7)	0.0033 (6)	0.0037 (6)	−0.0018 (6)
C5	0.0200 (8)	0.0245 (8)	0.0224 (7)	0.0032 (6)	0.0054 (6)	0.0052 (6)
C6	0.0214 (8)	0.0290 (9)	0.0195 (7)	0.0020 (6)	0.0040 (6)	−0.0033 (6)
C7	0.0158 (7)	0.0215 (8)	0.0198 (7)	0.0012 (6)	0.0042 (5)	0.0012 (6)
C8	0.0174 (7)	0.0190 (8)	0.0235 (7)	0.0005 (6)	0.0005 (6)	−0.0010 (6)
C9	0.0172 (7)	0.0161 (7)	0.0199 (7)	−0.0012 (6)	0.0007 (5)	−0.0024 (6)
C10	0.0220 (8)	0.0197 (7)	0.0216 (7)	0.0023 (6)	0.0009 (6)	0.0018 (6)
C11	0.0246 (8)	0.0201 (8)	0.0206 (7)	−0.0005 (6)	0.0034 (6)	0.0015 (6)
C12	0.0173 (7)	0.0205 (8)	0.0202 (7)	−0.0034 (6)	0.0034 (6)	−0.0044 (6)
C13	0.0177 (7)	0.0211 (8)	0.0253 (8)	0.0014 (6)	−0.0012 (6)	0.0015 (6)
C14	0.0197 (7)	0.0186 (7)	0.0245 (7)	−0.0015 (6)	0.0011 (6)	0.0042 (6)

### Geometric parameters (Å, º)

**Table:**

Br1—C12	1.9014 (15)	C5—H5A	0.96 (2)
O1—C8	1.2045 (19)	C5—H5B	0.98 (2)
O2—C8	1.3438 (18)	C6—H6A	0.95 (2)
O2—C7	1.4454 (18)	C6—H6B	0.96 (2)
C1—C2	1.514 (2)	C7—H7	0.985 (18)
C1—C7	1.540 (2)	C8—C9	1.491 (2)
C1—C6	1.556 (2)	C9—C14	1.392 (2)
C1—H1	0.979 (19)	C9—C10	1.394 (2)
C2—C3	1.332 (3)	C10—C11	1.387 (2)
C2—H2	0.95 (2)	C10—H10	0.95 (2)
C3—C4	1.512 (2)	C11—C12	1.386 (2)
C3—H3	0.95 (2)	C11—H11	0.95 (2)
C4—C7	1.534 (2)	C12—C13	1.385 (2)
C4—C5	1.557 (2)	C13—C14	1.385 (2)
C4—H4	0.97 (2)	C13—H13	0.94 (2)
C5—C6	1.550 (2)	C14—H14	0.94 (2)
			
C8—O2—C7	116.34 (12)	C1—C6—H6B	109.7 (12)
C2—C1—C7	97.94 (12)	H6A—C6—H6B	107.3 (16)
C2—C1—C6	106.86 (13)	O2—C7—C4	109.97 (12)
C7—C1—C6	100.89 (12)	O2—C7—C1	113.61 (12)
C2—C1—H1	116.7 (11)	C4—C7—C1	94.61 (12)
C7—C1—H1	116.6 (11)	O2—C7—H7	107.9 (10)
C6—C1—H1	115.3 (11)	C4—C7—H7	114.6 (11)
C3—C2—C1	107.73 (14)	C1—C7—H7	115.8 (11)
C3—C2—H2	127.0 (13)	O1—C8—O2	124.19 (14)
C1—C2—H2	124.9 (13)	O1—C8—C9	123.57 (14)
C2—C3—C4	107.94 (14)	O2—C8—C9	112.24 (13)
C2—C3—H3	129.8 (13)	C14—C9—C10	119.66 (14)
C4—C3—H3	122.1 (13)	C14—C9—C8	117.47 (14)
C3—C4—C7	98.17 (12)	C10—C9—C8	122.87 (14)
C3—C4—C5	106.87 (13)	C11—C10—C9	120.26 (14)
C7—C4—C5	100.92 (12)	C11—C10—H10	120.3 (13)
C3—C4—H4	117.4 (12)	C9—C10—H10	119.4 (13)
C7—C4—H4	116.1 (12)	C12—C11—C10	118.78 (15)
C5—C4—H4	114.8 (12)	C12—C11—H11	122.2 (13)
C6—C5—C4	103.10 (12)	C10—C11—H11	119.0 (13)
C6—C5—H5A	112.8 (12)	C13—C12—C11	122.07 (15)
C4—C5—H5A	111.4 (12)	C13—C12—Br1	118.94 (12)
C6—C5—H5B	110.7 (12)	C11—C12—Br1	118.99 (12)
C4—C5—H5B	110.2 (11)	C12—C13—C14	118.46 (14)
H5A—C5—H5B	108.6 (16)	C12—C13—H13	120.9 (12)
C5—C6—C1	103.23 (12)	C14—C13—H13	120.6 (12)
C5—C6—H6A	114.0 (12)	C13—C14—C9	120.74 (15)
C1—C6—H6A	111.2 (12)	C13—C14—H14	120.0 (13)
C5—C6—H6B	111.4 (12)	C9—C14—H14	119.2 (13)
			
C7—C1—C2—C3	−34.61 (16)	C2—C1—C7—C4	52.53 (13)
C6—C1—C2—C3	69.37 (17)	C6—C1—C7—C4	−56.44 (13)
C1—C2—C3—C4	0.40 (18)	C7—O2—C8—O1	1.3 (2)
C2—C3—C4—C7	34.14 (16)	C7—O2—C8—C9	−178.68 (12)
C2—C3—C4—C5	−69.96 (16)	O1—C8—C9—C14	8.7 (2)
C3—C4—C5—C6	66.21 (15)	O2—C8—C9—C14	−171.27 (13)
C7—C4—C5—C6	−35.90 (15)	O1—C8—C9—C10	−171.07 (16)
C4—C5—C6—C1	0.34 (15)	O2—C8—C9—C10	8.9 (2)
C2—C1—C6—C5	−66.69 (16)	C14—C9—C10—C11	−1.8 (2)
C7—C1—C6—C5	35.16 (15)	C8—C9—C10—C11	178.00 (14)
C8—O2—C7—C4	−164.96 (12)	C9—C10—C11—C12	0.1 (2)
C8—O2—C7—C1	90.43 (15)	C10—C11—C12—C13	1.6 (2)
C3—C4—C7—O2	−169.53 (12)	C10—C11—C12—Br1	−177.29 (12)
C5—C4—C7—O2	−60.49 (15)	C11—C12—C13—C14	−1.5 (2)
C3—C4—C7—C1	−52.35 (13)	Br1—C12—C13—C14	177.42 (12)
C5—C4—C7—C1	56.70 (13)	C12—C13—C14—C9	−0.3 (2)
C2—C1—C7—O2	166.68 (12)	C10—C9—C14—C13	1.9 (2)
C6—C1—C7—O2	57.71 (15)	C8—C9—C14—C13	−177.88 (14)

### Intermolecular close contacts.*

**Table:**

Contact	Distance (Å)	Contact	Distance (Å)
Br1^i^···C5^vi^	3.707 (2)	Br1^i^···H7^iv^	3.10 (2)
Br1^i^···C6^vi^	3.676 (2)	O1^i^···H6B^v^	2.56 (2)
H11^i^···O1^ii^	2.66 (2)	H14^i^···C11^v^	2.84 (2)

* [symmetry codes: (ii) 1-*x*, -1/2+*y*, 1/2 -*z*; (iv) 1-*x*, 1-*y*, -*z*; (v) *x*, 3/2-*y*, -1/2+*z*; (vi) 1-*x*, 1-*y*, 1-*z*]

### Interplanar angles (°).

**Table:**

Structure	1 : 2	1 : 3	2 : 3	2 : 4
1	124.5 (1)	122.7 (1)	112.8 (1)	6 (1)
2	121.2 (1)	125.5 (1)	113.3 (1)	

### Intramolecular contact comparison.

**Table:**

Contact	Structure 1 Distance (Å)	Structure 2 Distance (Å)
C2···C7	2.304 (2)	2.342 (2)
C3···C7	2.302 (2)	2.343 (3)
C5···C7	2.384 (2)	2.381 (3)
C6···C7	2.387 (2)	2.379 (3)
C2···C6	2.466 (2)	2.496 (3)
C3···C5	2.465 (2)	2.495 (3)
H3B···H5A		2.35 (3)
H2B···H6A		2.40 (3)

## References

[bb1] Altomare, A., Burla, M. C., Camalli, M., Cascarano, G. L., Giacovazzo, C., Guagliardi, A., Moliterni, A. G. G., Polidori, G. & Spagna, R. (1999). *J. Appl. Cryst.* **32**, 115–119.

[bb2] Chow, T. J. (1998). *J. Phys. Org. Chem.* **11**, 871–878.

[bb3] Coots, R. J. (1983). PhD Dissertation, Chemistry Department, University of Utah, USA.

[bb4] Farrugia, L. J. (1997). *J. Appl. Cryst.* **30**, 565.

[bb5] Farrugia, L. J. (1999). *J. Appl. Cryst.* **32**, 837–838.

[bb6] Jones, P. G., Kirby, A. J. & Percy, J. M. (1992). *Acta Cryst.* C**48**, 829–832.

[bb7] Lloyd, B. A., Arif, A. M. & Allred, E. L. (2000). *Acta Cryst.* C**56**, 1377–1379. 10.1107/s0108270100011185 11077305

[bb8] Lloyd, B. A., Arif, A. M., Coots, R. J. & Allred, E. L. (1995). *Acta Cryst.* C**51**, 2059–2062.

[bb9] Lloyd, B. A., Ericson, C., Arif, A. M. & Allred, E. L. (1993). *Acta Cryst.* C**49**, 257–261.

[bb10] Macdonald, A. C. & Trotter, J. (1965). *Acta Cryst.* **19**, 456–463.

[bb11] Nonius (1998). *COLLECT* Nonius BV, Delft, The Netherlands.

[bb12] Otwinowski, Z. & Minor, W. (1997). *Methods in Enzymology*, Vol. 276, *Macromolecular Crystallography*, Part A, edited by C. W. Carter Jr & R. M. Sweet, pp. 307–326. New York: Academic Press.

[bb13] Sheldrick, G. M. (2008). *Acta Cryst.* A**64**, 112–122. 10.1107/S0108767307043930 18156677

[bb14] Spek, A. L. (2009). *Acta Cryst.* D**65**, 148–155. 10.1107/S090744490804362X PMC2631630 19171970

[bb15] Westrip, S. P. (2010). *J. Appl. Cryst.* **43**, 920–925.

